# Prevalence, characteristics and mortality of cancer patients undergoing pericardiocentesis in the United States between 2004 and 2017

**DOI:** 10.1002/cam4.5373

**Published:** 2022-10-20

**Authors:** Andrija Matetic, Bonnie Ky, Eric H. Yang, Phyo K. Myint, Muhammad Rashid, Shelley Zieroth, Timir K. Paul, Ayman Elbadawi, Mamas A. Mamas

**Affiliations:** ^1^ Department of Cardiology University Hospital of Split Split Croatia; ^2^ Keele Cardiovascular Research Group Keele University Keele UK; ^3^ Cardiovascular Division, Department of Medicine Perelman School of Medicine of the University of Pennsylvania Philadelphia Pennsylvania USA; ^4^ Abramson Cancer Center, Perelman School of Medicine University of Pennsylvania Philadelphia Pennsylvania USA; ^5^ UCLA Cardio‐Oncology Program, Division of Cardiology, Department of Medicine University of California Los Angeles California USA; ^6^ Aberdeen Cardiovascular & Diabetes Centre University of Aberdeen Aberdeen UK; ^7^ Section of Cardiology, Department of Medicine University of Manitoba Winnipeg Manitoba Canada; ^8^ Department of Cardiovascular Sciences University of Tennessee at Nashville Nashville Tennessee USA; ^9^ Division of Cardiology University of Texas Southwestern Medical Center Dallas Texas USA

**Keywords:** cancer, characteristics, outcomes, pericardiocentesis, prevalence

## Abstract

**Background:**

Pericardiocentesis is undertaken in patients with cancer for diagnostic and therapeutic purposes. However, there are limited data on the frequency, characteristics and mortality of patients with different cancers undergoing pericardiocentesis.

**Methods:**

All hospitalisations of adult cancer patients (≥18 years) in the US National Inpatient Sample between January 2004 and December 2017 were included. The cohort was stratified by discharge code of pericardiocentesis and cancer, using the International Classification of Diseases. The prevalence of pericardiocentesis, patient characteristics, cancer types and in‐hospital all‐cause mortality were analysed between cancer patients undergoing pericardiocentesis versus not.

**Results:**

A total of 19,773,597 weighted cancer discharges were analysed, out of which 18,847 (0.1%) underwent pericardiocentesis. The most common cancer types amongst the patients receiving pericardiocentesis were lung (51.3%), haematological (15.9%), breast (5.4%), mediastinum/heart (3.2%), gastroesophageal (2.2%) and female genital cancer (1.8%), whilst ‘other’ cancer types were present in 20.2% patients. Patients undergoing pericardiocentesis had significantly higher mortality (15.6% vs. 4.2%, *p* < 0.001) compared to their counterparts. The presence of metastatic disease (aOR 2.67 95% CI 1.79–3.97), weight loss (aOR 1.48 95% CI 1.33–1.65) and coagulopathy (aOR 3.22 95% CI 1.63–6.37) were each independently associated with higher mortality in patients who underwent pericardiocentesis.

**Conclusion:**

Pericardiocentesis is an infrequent procedure in cancer patients and is most commonly performed in patients with lung, haematological and breast cancer. Cancer patients undergoing pericardiocentesis have increased mortality, irrespective of the underlying cancer type.

## Graphical abstract

Pericardiocentesis is most commonly performed in patients with lung, haematological and breast cancer. Cancer patients undergoing pericardiocentesis have increased mortality. There are different predictors of increased mortality in patients undergoing pericardiocentesis, including presence of metastatic disease, weight loss and coagulopathy.

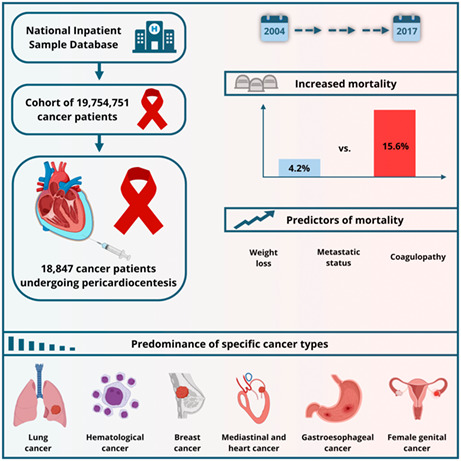

## 1 INTRODUCTION

Pericardial effusion is a common occurrence in patients with known or suspected cancer with diagnostic and therapeutic implications. It is estimated that 25%–46% of overall patients undergoing pericardiocentesis have malignant pericardial effusion.^1^, ^2^, ^3^ Pericardial effusion can also complicate active cancer treatment.^4^ Pericardial effusion varies in clinical presentation, prevalence and effusion volume amongst different cancer diagnoses, which may drive decision making around the need for pericardiocentesis.

Pericardiocentesis is more complex in patients with cancer, and some patient characteristics such as metastatic status, cancer type and comorbidities have an impact on the procedural complications.^5^ There are limited data around differences in the utilisation of pericardiocentesis amongst real‐world cancer populations, particularly when comparing across different cancer types, and whether there are differences in patient characteristics and clinical outcomes. Few studies reported overall worse outcomes in cancer patients undergoing pericardiocentesis compared to their non‐cancer counterparts.^2^, ^6^ It was also suggested that lung cancer patients undergoing pericardiocentesis have the worst outcomes,^1^, ^5^ whilst patients with haematological diseases have better outcomes compared to those with non‐haematologic malignancy.^5^, ^7^ However, existing literature includes single‐centre or sub‐analyses with small sample sizes warranting further large‐scale studies.^1^, ^2^, ^5^, ^6^, ^7^, ^8^

This study, therefore, aimed to determine the overall utilisation of pericardiocentesis in a real‐world national cancer population over time. It aimed to determine the most prevalent cancer types undergoing pericardiocentesis, including their characteristics and mortality. Finally, it aimed to determine the predictors of mortality amongst cancer patients undergoing pericardiocentesis.

## 2 METHODS

The National Inpatient Sample (NIS) database represents the largest healthcare database of routinely collected data in the United States (US) comprising anonymised discharge data from >7 million hospitalisations yearly. It includes data from approximately 20% of inpatient hospital stays (excluding rehabilitation or long‐term acute care hospitals) from all US regions.^9^ It was created by the Agency for Healthcare Research and Quality (AHRQ) under the Healthcare Cost and Utilisation Project (HCUP) to produce the US nationally representative estimates of healthcare resource utilisation, access, quality, and outcomes.^9^ It is fully based on retrospective data, and starting from 1988, it obtains data through hospital discharge records from all hospitals participating in the HCUP. Collected data are being aggregated to form a national database from which retrospective research analyses can be performed.

The NIS database has several advantages for large observational analyses, including anonymised data, sufficiently powered population samples, coverage of a long period of time, and a very broad capture of comorbidities. Furthermore, due to its reliance on the *International classification of Diseases* system, including the ninth revision (ICD‐9) and 10th revision (ICD‐10), means that there is a possibility of external validation of the study findings.^9^

## 3 STUDY SAMPLE

This study included all adult hospitalisations (≥18 years) with a cancer diagnosis between January 2004 and December 2017. The study sample was derived using the discharge diagnostic codes for ‘cancer’ (any diagnostic priority). The ICD‐9 codes were used for the initial study period (January 2004–September 2015), whilst the ICD‐10 codes were used for the remaining study period (October 2015–December 2017), as described in Table S1.

The study sample was further stratified according to the discharge procedure codes for ‘pericardiocentesis’ and discharge diagnostic codes for different cancer types (any diagnostic priority for both) (Table S1). The most common cancer types undergoing pericardiocentesis were of particular interest (lung cancer, haematological cancer, breast cancer, mediastinal and heart cancer, gastroesophageal cancer, female genital cancer, and ‘other’ cancer) and were additionally investigated including their characteristics and outcomes (Table S1). The ICD‐9 and ICD‐10 coding systems were carefully used to detect the diagnoses, conditions or procedures of interest. Other variables that could be relevant to the outcomes were also captured from the NIS, including ‘weekend admission’ and hospital‐related factors (‘hospital bed size,’ ‘hospital region’ and ‘hospital location/teaching status’). ‘Weekend admission’ variable is an indicator of whether the admission day is on the weekend and is calculated from the admission date. ‘Hospital bed size’ variable refers to the number of short‐term acute hospital beds and is specific to the hospital's location and teaching status.^9^ Economic analysis was not the focus of the study which is why hospitalisation charges were not adjusted for inflation.

Cases excluded due to missing data represented 2.3% (*n* = 469,296) of the original dataset (Figure S1). This observational study was appraised according to the *Strengthening The Reporting of OBservational Studies in Epidemiology (STROBE*) (Appendix A).

## 4 OBJECTIVES/AIMS

We aimed to evaluate the prevalence of pericardiocentesis and patient characteristics amongst cancer cohorts and different cancer types. We also aimed to examine the in‐hospital all‐cause mortality stratified by the utilisation of pericardiocentesis and cancer type, as well as the predictors of mortality in the pericardiocentesis cohort.

### 4.1 Statistical analysis

Data were expressed as numbers (percentages) for categorical data and as median (interquartile range) for continuous data. Categorical variables were analysed using a Chi‐square test, whilst continuous variables were analysed with the Kruskal–Wallis test. Binomial multivariable logistic regression analysis was conducted to determine the association of different variables with all‐cause mortality and was expressed as adjusted odds ratios (aOR) with 95% confidence intervals (95% CI). The following variables were assessed due to their potential association with all‐cause mortality: Age, sex, metastatic status, weight loss, anaemias, coagulopathy, thrombocytopenia, congestive heart failure, atrial fibrillation, diabetes, arterial hypertension and chronic renal failure. All analyses were weighted using the provided discharge weights, and hierarchical multilevel modelling was used to account for the clustering/nesting of observations, as recommended by HCUP. Statistical significance was defined at a level of *p* < 0.05. SPSS 25 software (IBM Corp) and Stata MP version 16.0 (StataCorp) were used for statistical analysis.

## 5 RESULTS

### 5.1 Baseline characteristics

A total of 19,773,597 weighted hospitalisations with a cancer diagnosis were included, out of which 18,847 (0.1%) underwent pericardiocentesis (Figure S1). Patients undergoing pericardiocentesis were more often admitted during the weekend (19.0% vs. 10.3%, *p* < 0.001) and had a higher proportion of metastatic disease (20.9% vs. 11.1%, *p* < 0.001), as well as comorbidities such as anaemias (32.0% vs. 22.4%, *p* < 0.001), atrial fibrillation (29.5% vs. 8.8%, *p* < 0.001), congestive heart failure (11.6% vs. 5.6%, *p* < 0.001), coagulopathy (11.2% vs. 6.1%, *p* < 0.001), thrombocytopenia (6.9% vs. 4.9%, *p* < 0.001), electrolyte disorders (43.2% vs. 23.0%, *p* < 0.001) and weight loss (19.7% vs. 10.2%, *p* < 0.001) (Table 1).

**TABLE 1:** Baseline characteristics of cancer patients based on the utilisation of pericardiocentesis

Characteristics	Cancer patients		*p*‐Value
	Not undergoing pericardiocentesis (99.9%)	Undergoing pericardiocentesis (0.1%)	
Number of hospitalisations	19,754,751	18,847	
Age (years), median (IQR)	62 (50, 73)	59 (50, 69)	<0.001
Female sex, %	53.6	52.3	<0.001
Race/ethnicity, %			<0.001
White	69.6	68.4	
Black	14.2	14.3	
Hispanic	9.2	9.2	
Asian or Pacific Islander	3.2	5.2	
Native American	0.4	0.5	
Other	3.4	2.5	
Weekend admission, %	10.3	19.0	<0.001
Primary expected payer, %			<0.001
Medicare	44.8	37.3	
Medicaid	11.2	15.8	
Private Insurance	37.5	38.4	
Self‐pay	3.0	4.9	
No charge	0.4	0.4	
Other	3.1	3.2	
Median household income (percentile), %			<0.001
0–25th	26.4	26.6	
26th–50th	24.9	24.9	
51st–75th	24.6	25.0	
76th–100th	24.1	23.4	
Diabetes Mellitus	19.8	15.1	<0.001
Arterial hypertension	44.8	38.3	<0.001
Anaemias	22.4	32.0	<0.001
Atrial fibrillation	8.8	29.5	<0.001
Rheumatoid arthritis/Collagen disease	1.9	2.2	0.014
Congestive heart failure	5.6	11.6	<0.001
Valvular disease	3.3	3.7	<0.001
Peripheral vascular disorders	3.7	4.2	<0.001
Hypothyroidism	10.3	9.4	<0.001
Chronic pulmonary disease	18.1	31.1	<0.001
Coagulopathy	6.1	11.2	<0.001
Thrombocytopenia	4.9	6.9	<0.001
Depression	9.3	8.8	<0.001
Liver disease	3.6	3.6	0.434
Chronic renal failure	7.6	8.7	<0.001
Alcohol abuse	2.5	2.8	<0.001
Drug abuse	1.4	2.6	<0.001
Fluid and electrolyte disorders	23.0	43.2	<0.001
Weight loss	10.2	19.7	<0.001
Obesity	10.9	7.3	<0.001
Metastatic cancer	11.1	20.9	<0.001
Bed size of hospital, %			<0.001
Small	12.3	10.7	
Medium	24.0	19.2	
Large	63.7	70.0	
Hospital Region, %			<0.001
Northeast	21.7	19.4	
Midwest	21.7	25.0	
South	38.2	35.7	
West	18.5	19.8	
Location/teaching status of hospital, %			<0.001
Rural	5.7	2.6	
Urban non‐teaching	24.4	20.9	
Urban teaching	69.9	76.5	

Abbreviation: IQR, interquartile range.

### 5.2 Prevalence and characteristics of different cancer types

**FIGURE 1:**
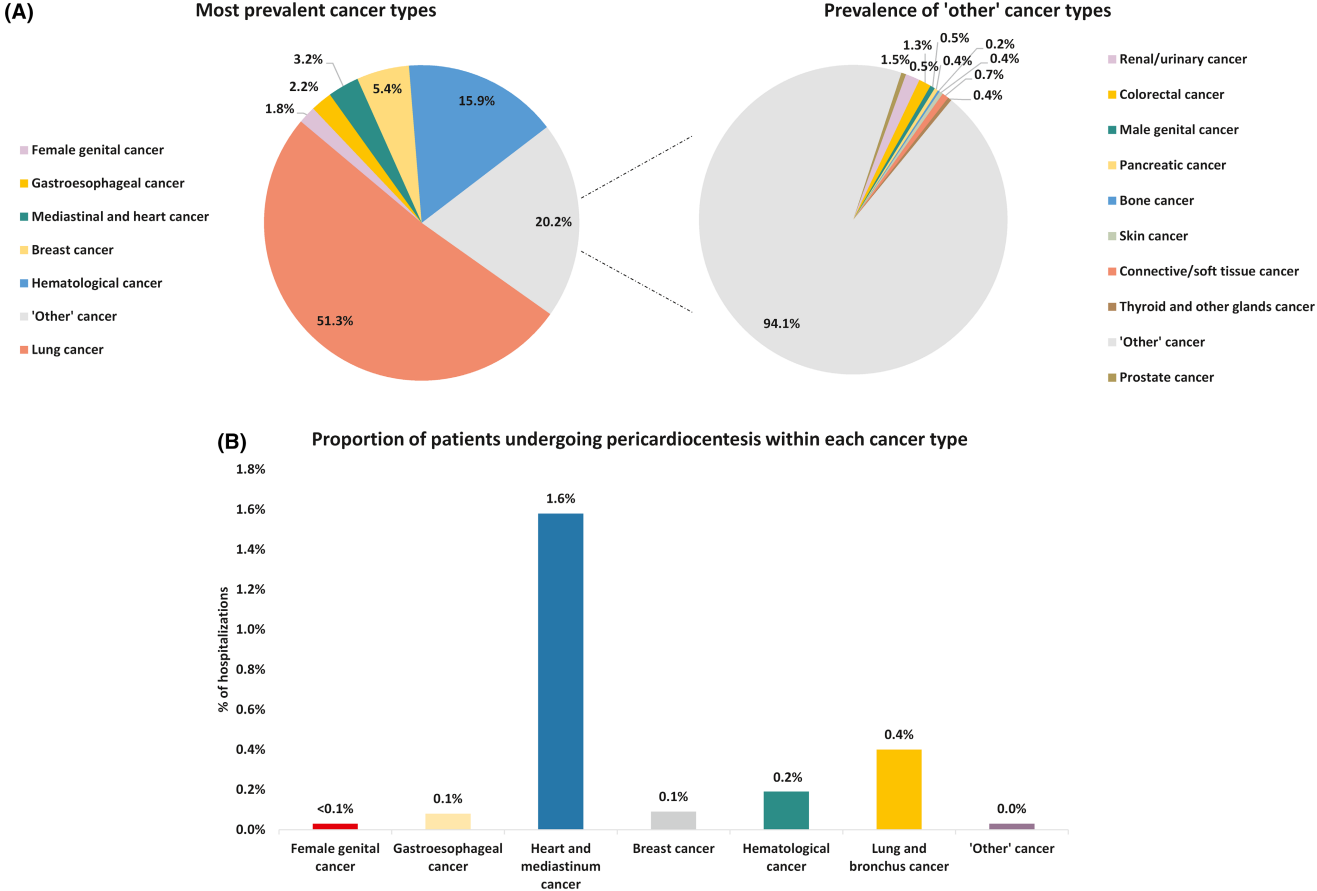
Prevalence of different cancer types in the study cohort: (A) Patients undergoing pericardiocentesis; (B) Patients not undergoing pericardiocentesis.

The most common cancer types amongst the patients receiving pericardiocentesis were lung cancer (51.3%), haematological cancer (15.9%), breast cancer (5.4%), mediastinum and heart cancer (3.2%), gastroesophageal cancer (2.2%) and female genital cancer (1.8%), whilst ‘other’ cancer types were present in 20.2% patients (Figure 1A). These findings were consistent when looking at the yearly distribution of different cancer types across the study period (Figure S2A). When looking at the proportion of patients undergoing pericardiocentesis within each cancer type, the highest proportion was observed in the mediastinum and heart cancer (1.6%), followed by lung and bronchus cancer (0.4%) and haematological cancer (0.2%), whilst pericardiocentesis was undertaken in <0.1% of patients in other cancer types (Figure 1B).

When comparing groups based on the receipt of pericardiocentesis in the most common cancer types, patients undergoing pericardiocentesis were overall younger and had a higher proportion of metastatic disease (*p* < 0.05) (Table 2). The differences in major comorbidities were generally consistent with the findings in the overall cohort (Table 2).

**TABLE 2:** Baseline characteristics of cancer patients based on the utilisation of pericardiocentesis across the most common cancer types (requiring pericardiocentesis)

Characteristics	Lung cancer			Haematological cancer			Breast cancer			Mediastinal and heart cancer			Gastroesophageal cancer			Female genital cancer			‘Other’ cancer		
	No pericardiocentesis (99.60%)	Pericardiocentesis (0.40%)	*p*‐Value	No pericardiocentesis (99.81%)	Pericardiocentesis (0.19%)	*p*‐Value	No pericardiocentesis (99.91%)	Pericardiocentesis (0.09%)	*p*‐Value	No pericardiocentesis (98.42%)	Pericardiocentesis (1.58%)	*p*‐Value	No pericardiocentesis (99.92%)	Pericardiocentesis (0.08%)	*p*‐Value	No pericardiocentesis (99.97%)	Pericardiocentesis (0.03%)	*p*‐Value	No pericardiocentesis (99.97%)	Pericardiocentesis (0.03%)	*p*‐Value
Number of hospitalizations	2,335,650	9488		1,568,973	3005		1,102,239	999		34,695	557		510,364	414		1,157,306	332		13,045,524	4051	
Age (years), median (IQR)	68 (60, 76)	62 (54, 70)	<0.001	64 (50, 75)	45 (25, 64)	<0.001	60 (49, 71)	56 (47, 65)	<0.001	58 (41, 70)	55 (30, 67)	0.002	66 (57, 76)	59 (52, 66)	<0.001	62 (52, 71)	59 (51, 68)	<0.001	61 (48, 72)	59 (48, 70)	<0.001
Female sex, %	48.3	49.4	0.057	43.0	43.1	0.141	99.1	100.0	0.033	40.9	42.9	0.506	31.0	20.8	<0.001	/	/	/	49.1	51.0	<0.001
Race/ethnicity, %																					
White	77.1	69.8	<0.001	69.2	64.3	<0.001	67.1	62.8	<0.001	65.3	72.5	0.001	63.8	65.2	<0.001	69.6	63.0	<0.001	68.7	70.0	<0.001
Black	12.7	15.4		12.7	5.1		16.3	16.3		14.0	15.0		14.9	8.7		13.0	18.5		14.7	9.3	
Hispanic	4.7	5.8		14.1	14.1		9.1	12.8		10.8	10.0		11.6	21.7		9.9	11.1		9.6	11.9	
Asian or Pacific Islander	2.8	6.1		2.9	3.0		3.6	4.7		4.9	<0.1		5.5	4.4		3.7	3.7		3.2	5.7	
Native American	0.4	0.3		0.4	1.0		0.4	<0.1		0.6	2.5		0.5	<0.1		0.5	<0.1		0.5	0.5	
Other	2.3	2.5		3.9	2.5		3.5	3.5		4.3	<0.1		3.7	<0.1		3.4	3.7		3.5	2.6	
Weekend admission, %	14.8	19.8	<0.001	16.1	17.3	<0.001	7.3	15.7	<0.001	12.4	14.3	<0.001	14.2	29.2	<0.001	8.7	21.4	<0.001	8.9	19.1	<0.001
Primary expected payer, %																					
Medicare	60.1	42.4	<0.001	46.4	23.1	<0.001	36.0	28.1	<0.001	35.7	28.6	<0.001	51.1	32.7	<0.001	43.3	35.7	<0.001	42.4	41.0	<0.001
Medicaid	10.2	15.9		12.9	21.8		13.1	16.9		16.4	16.7		11.9	12.8		12.9	21.4		10.7	9.2	
Private Insurance	23.6	33.6		34.0	45.3		46.1	51.7		40.0	38.1		30.1	42.6		36.8	25.0		40.3	42.2	
Self‐pay	2.7	4.8		3.0	4.0		2.0	2.3		4.2	14.3		3.5	4.6		3.6	14.3		3.1	4.8	
No charge	0.3	0.6		0.3	0.1		0.3	<0.1		0.3	<0.1		0.4	<0.1		0.6	<0.1		0.4	0.4	
Other	3.1	2.7		3.4	5.8		2.5	1.1		3.3	2.4		2.9	7.3		2.8	3.6		3.2	2.4	
Median household income (percentile), %																					
0–25th	29.7	31.0	<0.001	25.6	21.4	<0.001	23.5	21.0	0.693	26.4	38.1	0.006	28.1	30.4	0.003	26.4	28.6	0.007	26.1	19.2	<0.001
26th–50th	26.5	22.9		24.7	26.8		23.0	32.2		23.5	23.8		24.8	23.9		24.7	28.6		24.9	24.9	
51st–75th	23.7	24.9		25.1	27.3		25.0	23.0		24.1	19.1		24.1	16.8		24.9	28.6		24.8	25.7	
76th–100th	21.0	21.2		24.6	24.5		28.6	24.1		26.0	19.1		23.0	29.0		24.0	14.3		24.4	30.2	
Diabetes Mellitus	21.1	15.0	<0.001	19.6	13.8	<0.001	15.8	12.4	0.001	13.1	7.1	<0.001	20.3	10.4	<0.001	18.6	18.1	0.808	17.0	13.7	<0.001
Arterial hypertension	52.1	42.6	<0.001	43.2	31.7	<0.001	40.6	36.7	0.013	39.1	28.7	<0.001	48.8	33.5	<0.001	46,4	38.5	0.004	43.8	35.2	<0.001
Anaemias	22.3	31.1	<0.001	37.8	32.0	0.619	14.0	21.4	<0.001	20.8	42.9	<0.001	32.6	33.1	0.819	20.6	41.0	<0.001	18.8	27.5	<0.001
Rheumatoid arthritis/Collagen disease	2.8	2.6	0.001	2.3	1.4	0.637	1.8	1.2	0.876	2.3	0.3	0.098	1.3	2.2	0.125	2.0	1.6	0.573	1.4	1.8	0.009
Obesity	5.0	3.9	<0.001	6.0	7.3	0.003	7.6	4.8	0.001	6.4	3.5	0.005	5.2	3.7	0.163	16.2	9.0	<0.001	8.0	5.8	<0.001
Congestive heart failure	8.6	11.1	<0.001	8.7	13.7	<0.001	3.8	9.9	<0.001	5.6	2.5	0.034	6.9	19.8	<0.001	4.1	12.1	<0.001	4.6	12.6	<0.001
Atrial fibrillation	15.9	33.0	<0.001	11.1	19.1	<0.0011	4.4	18.0	<0.001	13.7	26.2	<0.001	12.6	25.1	<0.001	5.2	16.0	<0.001	6.4	27.0	<0.001
Valvular disease	4.0	4.4	0.128	4.1	3.8	0.094	2.6	2.6	0.543	3.5	<0.1	0.001	3.7	3.4	0.752	3.1	<0.1	0.001	3.1	3.5	0.230
Peripheral vascular disorders	8.2	6.1	<0.001	3.4	1.4	0.027	1.3	1.2	0.239	4.2	2.5	0.035	4.4	2.4	0.056	1.6	<0.1	0.021	2.6	3.0	0.122
Hypothyroidism	10.8	9.1	<0.001	11.3	6.1	<0.001	12.1	4.9	0.021	8.1	15.0	0.068	7.4	15.6	<0.001	12.7	10.8	0.314	8.1	9.0	0.044
Chronic pulmonary disease	49.7	44.0	<0.001	13.9	13.2	0.059	12.3	17.3	<0.001	25.4	22.5	0.003	17.6	18.1	0.813	11.1	12.1	0.569	12.9	19.5	<0.001
Coagulopathy	5.2	7.6	<0.001	22.9	25.0	0.332	3.5	4.9	<0.001	6.7	10.0	<0.001	5.4	10.5	<0.001	2.9	11.8	<0.001	3.4	10.2	<0.001
Thrombocytopenia	4.4	5.0	<0.001	19.6	16.0	<0.001	2.9	2.3	<0.001	4.9	7.1	0.006	3.7	4.6	0.349	1.9	1.5	0.633	2.3	5.0	<0.001
Depression	11.4	11.2	0.014	10.4	3.3	<0.001	10.9	11.1	0.416	8.3	5.0	0.030	7.0	8.6	0.213	8.9	3.1	<0.001	6.9	7.1	0.604
Liver disease	2.6	4.0	<0.001	4.0	4.3	<0.001	1.9	2.5	0.002	2.6	<0.1	0.107	3.7	1.2	0.007	1.6	4.3	<0.001	3.0	2.2	0.001
Chronic renal failure	8.3	7.7	0.011	13.0	13.2	<0.001	3.7	3.6	0.084	4.5	5.0	0.148	6.5	8.6	0.090	4.4	4.6	0.798	5.1	8.3	<0.001
Alcohol abuse	4.1	4.0	0.001	1.6	0.9	0.086	0.7	1.2	0.916	2.4	0.9	0.023	4.6	3.4	0.245	0.6	1.6	0.033	2.0	2.3	0.170
Drug abuse	2.0	2.6	0.792	1.7	3.3	0.001	0.8	2.5	0.036	1.8	6.4	<0.001	1.2	2.4	0.033	0.7	<0.1	0.129	0.9	1.5	<0.001
Fluid and electrolyte disorders	28.2	41.0	<0.001	38.5	42.9	<0.001	13.3	46.9	<0.001	21.4	33.1	<0.001	35.1	56.5	<0.001	19.1	37.6	<0.001	16.7	41.1	<0.001
Weight loss	14.0	19.4	<0.0011	13.4	15.1	0.082	4.7	19.8	<0.001	9.0	10.0	0.405	24.9	31.3	0.003	6.1	10.6	0.001	6.2	13.1	<0.001
Metastatic cancer	14.1	29.0	<0.001	3.4	2.4	0.719	13.4	17.3	<0.001	13.8	19.9	<0.001	17.2	23.7	0.001	8.8	16.8	<0.001	10.8	19.4	<0.001
Bed size of hospital, %																					
Small	12.4	10.2	<0.001	12.3	9.9	<0.001	15.8	11.7	<0.001	8.5	8.3	0.274	10.3	5.7	0.022	9.0	4.8	<0.001	10.6	8.8	<0.001
Medium	25.0	19.6		20.7	19.9		26.2	21.6		20.9	18.0		22.1	23.4		20.2	11.5		22.4	18.0	
Large	62.7	70.3		67.0	70.2		57.9	66.7		70.6	73.8		67.6	70.9		70.8	83.7		67.0	73.2	
Hospital region, %																					
Northeast	21.5	19.0	<0.001	21.7	22.4	<0.001	25.1	25.5	<0.001	21.9	17.2	0.014	24.1	23.3	0.002	22.1	16.3	0.114	21.8	20.5	<0.001
Midwest	23.2	25.3		22.9	25.1		19.3	23.5		20.7	22.0		21.4	16.2		23.0	27.0		22.0	25.6	
South	41.3	35.9		37.3	31.4		36.5	29.4		38.5	37.4		35.9	34.5		35.7	36.1		37.5	30.7	
West	14.0	19.8		18.1	21.1		19.2	21.6		18.9	23.4		18.5	26.0		19.2	20.6		18.7	23.2	
Location/teaching status of hospital, %																					
Rural	7.5	3.5	<0.001	4.5	1.9	<0.001	6.5	7.8	<0.001	4.5	<0.1	<0.001	7.1	7.5	0.954	5.2	<0.1	0.001	7.4	3.5	<0.001
Urban non‐teaching	29.2	24.4		19.9	10.6		28.2	23.5		27.9	23.3		32.0	31.4		26.1	26.1		32.6	29.0	
Urban teaching	63.3	72.2		75.6	87.6		65.2	68.6		67.6	76.7		60.9	61.1		68.7	73.9		60.0	67.5	

Abbreviation: IQR, interquartile range.

### 5.3 All‐cause mortality and other clinical outcomes

**FIGURE 2:**
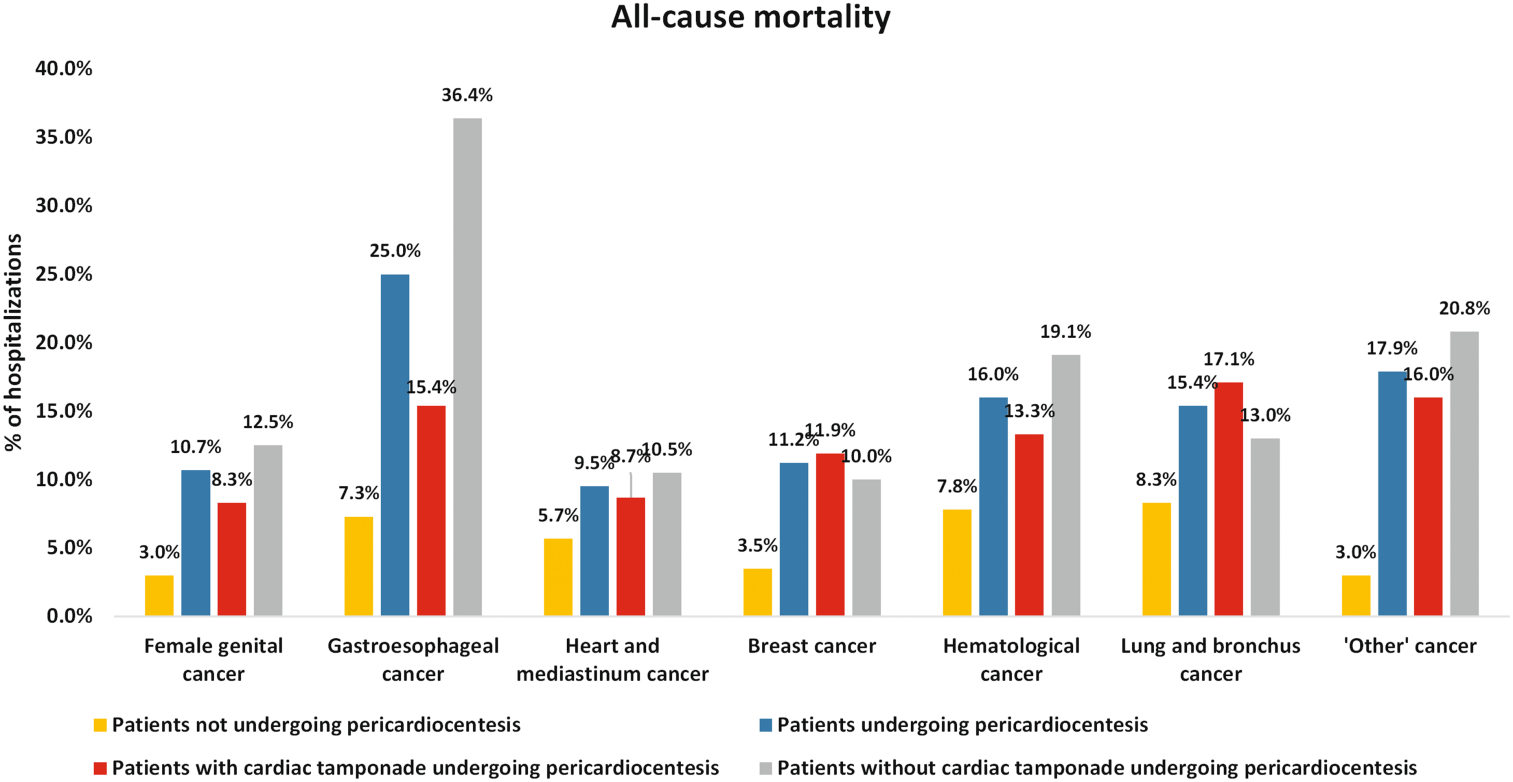
All‐cause mortality across the most common cancer types.

Patients undergoing pericardiocentesis had a significantly higher all‐cause mortality (15.6% vs. 4.2%, *p* < 0.001), longer length of stay (median of 9 vs. 4 days, *p* < 0.001) and increased total charges (median of 71,489 vs. 33,469 United States Dollars, *p* < 0.001) compared to their counterparts (Table 3). These findings were consistently present across the most common cancer types (Table 4 and Figure 2). When looking at the absolute rates of mortality in patients undergoing pericardiocentesis, it was the highest in patients with gastroesophageal cancer (25.0%), and the lowest in patients with heart and mediastinum cancer (9.5%) (Table 4 and Figure 2).

**TABLE 3:** Clinical outcomes of cancer patients based on the utilisation of pericardiocentesis

Characteristics	Cancer patients		*p*‐value
	Not undergoing pericardiocentesis (99.9%)	Undergoing pericardiocentesis (0.1%)	
All‐cause mortality	4.2	15.6	<0.001
Length of stay (days), median (IQR)	4 (2, 7)	9 (5, 14)	<0.001
Total charges (USD), median (IQR)	33,459 (18,069, 62,938)	71,489 (40,692, 133,669)	<0.001

Abbreviations: IQR, interquartile range; USD, United States Dollar.

**TABLE 4:** Clinical outcomes of cancer patients based on the utilisation of pericardiocentesis across the most common cancer types (requiring pericardiocentesis)

Characteristics	Lung cancer			Haematological cancer			Breast cancer			Mediastinal and heart cancer			Gastroesophageal cancer			Female genital cancer			‘Other’ cancer		
	No pericardiocentesis (99.60%)	Pericardiocentesis (0.40%)	*p*‐Value	No pericardiocentesis (99.81%)	Pericardiocentesis (0.19%)	*p*‐Value	No pericardiocentesis (99.91%)	Pericardiocentesis (0.09%)	*p*‐Value	No pericardiocentesis (98.42%)	Pericardiocentesis (1.58%)	*p*‐Value	No pericardiocentesis (99.92%)	Pericardiocentesis (0.08%)	*p*‐Value	No pericardiocentesis (99.97%)	Pericardiocentesis (0.03%)	*p*‐Value	No pericardiocentesis (99.97%)	Pericardiocentesis (0.03%)	*p*‐Value
All‐cause mortality	8.3	15.4	<0.001	7.8	16.0	<0.001	3.5	11.2	<0.001	5.7	9.5	0.001	7.3	25.0	<0.001	3.0	10.7	<0.001	3.0	17.9	<0.001
Length of stay (days), median (IQR)	5 (3, 9)	8 (5, 13)	<0.001	7 (3, 16)	12 (7, 22)	<0.001	2 (1, 4)	6 (4, 11)	<0.001	5 (3, 9)	11 (6, 15)	<0.001	7 (4, 11)	9 (5, 17)	<0.001	4 (2, 6)	8 (5, 14)	<0.001	3 (2, 7)	8 (5,14)	<0.001
Total charges (USD), median (IQR)	37,333 (19,208, 67,247)	66,859 (39,720, 115,214)	<0.001	52,908 (23,448, 128,475)	122,355 (61,738, 242, 687)	<0.001	26,328 (14,926, 48,633)	54,219 (32,388, 103,481)	<0.001	46,847 (25,729, 89,905)	88,121 (46,954, 192,221)	<0.001	45,171 (21,736, 90,823)	86,103 (36,707, 145,260)	<0.001	32,789 (18,886, 56,720)	62,768 (46,012, 121,681)	<0.001	31,953 (17,675, 58,686)	64,123 (36,517, 123,344)	<0.001

Abbreviation: IQR, interquartile range.

### 5.4 Sensitivity analysis based on cardiac tamponade

Cardiac tamponade was present in patients undergoing pericardiocentesis across all cancer types, with the highest prevalence in breast cancer (66.3%) and lowest prevalence in female genital cancer (42.9%) (Figure S3). All‐cause mortality was lower in patients with cardiac tamponade undergoing pericardiocentesis across all cancer types, except in those with breast cancer (11.9% vs. 10.0%) and lung/bronchus cancer (17.1% vs. 13.0%) when compared with patients undergoing pericardiocentesis without cardiac tamponade (Figure 2).

### 5.5 Predictors of all‐cause mortality

**FIGURE 3:**
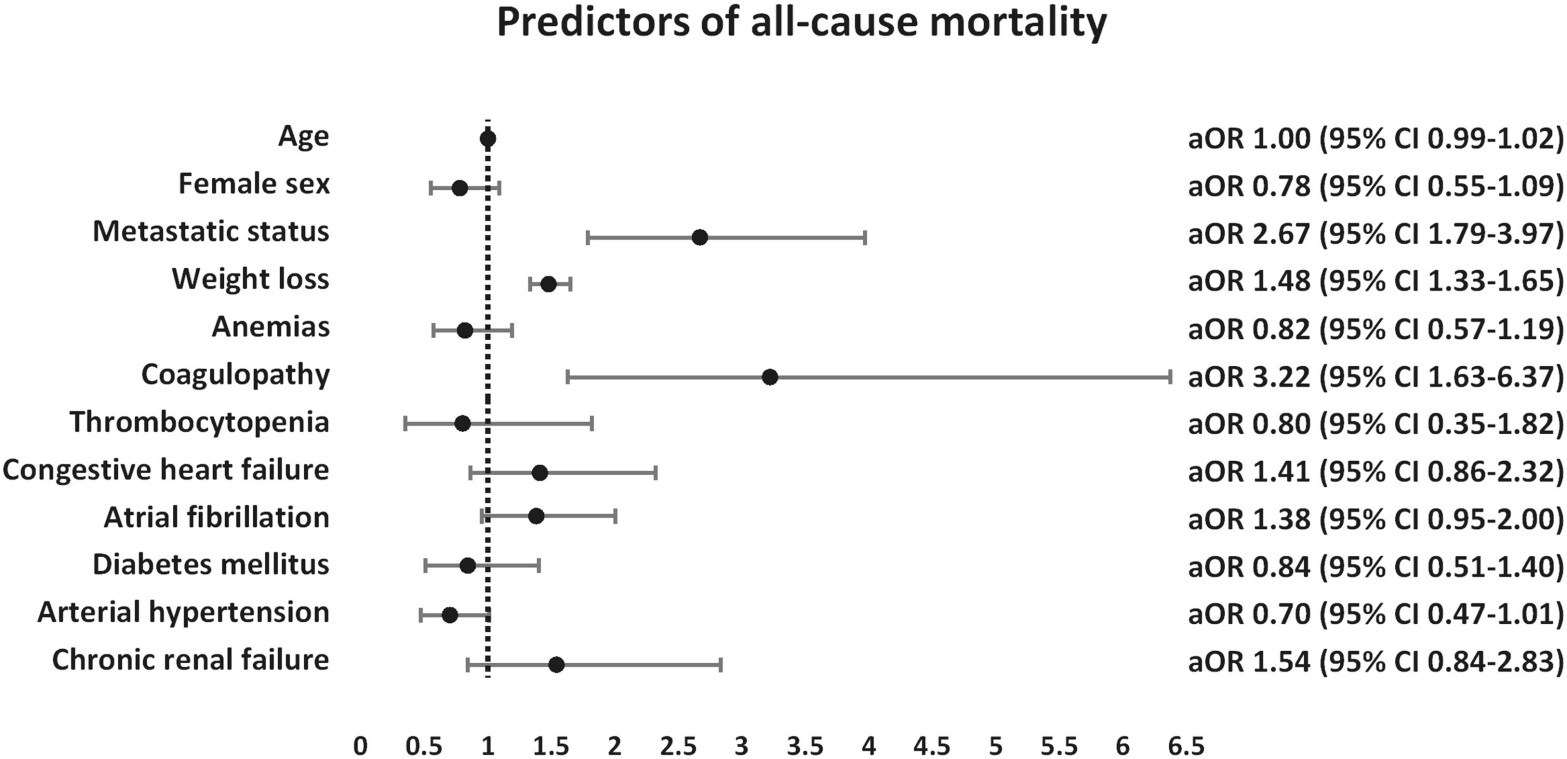
Predictors of all‐cause mortality in patients undergoing pericardiocentesis.

The presence of metastatic disease (aOR 2.67 95% CI 1.79–3.97), weight loss (aOR 1.48 95% CI 1.33–1.65) and coagulopathy (aOR 3.22 95% CI 1.63–6.37) was independently associated with all‐cause mortality in the pericardiocentesis cohort, whilst there was no association of age, sex, anaemias, thrombocytopenia, heart failure, atrial fibrillation, diabetes mellitus, hypertension and chronic renal failure with mortality in this group (*p* > 0.05) (Figure 3).

## 6 DISCUSSION

To the best of our knowledge, this is the largest cancer‐specific study to this date evaluating the prevalence, characteristics and outcomes of cancer patients undergoing pericardiocentesis. Its strengths further include a national‐level analysis and a comprehensive evaluation of the different cancer types. Several previous cohort studies evaluated cancer patients undergoing pericardiocentesis but included single‐centre analyses over a shorter period with substantially lower sample size.^1^, ^2^, ^5^, ^6^, ^7^, ^8^ This study offers several important findings. First, it revealed that pericardiocentesis is infrequently utilised in cancer cohorts covering only a minority of patients (~0.1%). Second, it is distinctively used amongst different cancer types, with the highest utilisation in the lung, haematological and breast cancer, followed by heart/mediastinum, gastroesophageal and female genital cancer. Third, this cohort has an increased prevalence of comorbidities that are considered to be higher risk in pericardiocentesis, such as anaemias, atrial fibrillation (due to anticoagulation), coagulopathy and thrombocytopenia.^5^, ^10^, ^11^ Fourth, cancer patients undergoing pericardiocentesis have increased mortality compared to other cancer patients admitted to hospitals and that overall mortality rates are dependent on the underlying cancer type. Finally, we identified independent predictors of increased mortality with metastatic status, weight loss and coagulopathy.

Pericardiocentesis is indicated for different diagnostic and therapeutic indications in the cancer population. Due to a strong association between cancer and pericardial effusion, it is more often undertaken than the general population and requires strict protocols to minimise the risk associated with the procedure.^11^ Previous studies have shown that cancer is an underlying cause of pericardial effusion in up to 46% of patients undergoing pericardiocentesis.^1^, ^2^, ^3^ Pericardial effusion may be associated with cancer metastases, but also with systemic cancer effects (hypoalbuminemia, impaired lymphatic drainage) or cancer treatments (i.e., immune checkpoint inhibitor therapy). The occurrence of pericardial effusion and subsequent utilisation of pericardiocentesis differs across cancer types. The present study showed that pericardiocentesis is most utilised in lung, haematological and breast cancer, followed by heart/mediastinum, gastroesophageal and female genital cancer. This is consistent with previous reports.^2^, ^5^, ^12^ All aforementioned cancer types could potentiate the development of pericardial effusion with direct or indirect mechanisms, such as serosal involvement,^13^ direct extensions with local inflammation and cellular toxicity,^14^ cancer‐induced cachexia and hypoalbuminemia,^15^ as well as lymphatic involvement with lymphedema.^14^ Furthermore, other determinants could additionally provoke pericardial effusion and increase the utilisation of pericardiocentesis, such as cancer treatment toxicity and opportunistic infections.^14^ High utilisation of pericardiocentesis in these cancer types is, therefore, not surprising.

One NIS‐based study investigated temporal trends and in‐hospital mortality of all‐comers undergoing pericardiocentesis over a period from 2007 to 2015.^16^ In this study, around 25% of patients had active cancer, and this was associated with increased in‐hospital mortality (OR 1.72; 95% CI 1.6–1.85).^16^ Importantly, the number of pericardiocentesis procedures increased over time, although there was no cancer‐focused analysis to evaluate specific trends.^16^ Another focused analysis of 212 cancer patients undergoing pericardiocentesis at the *MD Anderson Cancer Center* described the feasibility of percutaneous pericardiocentesis with no procedure‐related deaths.^5^ However, 1‐month (18%) and 2‐year mortality rates (61%) were substantially high and were associated with lung cancer, older age and severe grade 4 thrombocytopenia.^5^ Lung cancer patients undergoing pericardiocentesis were previously shown to have the highest mortality compared to other cancer types,^1^, ^5^, ^17^, ^18^ although this was not confirmed in the present study which revealed the highest mortality with gastroesophageal cancer. High recurrence (~25%) and 1‐year mortality rates (~55%) in cancer patients undergoing pericardiocentesis were also previously reported in a small Asian cohort study.^1^ Compared to non‐cancer patients undergoing pericardiocentesis, cancer patients undergoing pericardiocentesis were shown to have significantly increased in‐hospital and 1‐year mortality.^2^, ^6^ These findings are consistent with the present study, suggesting poor prognosis of cancer patients undergoing pericardiocentesis.

The high mortality rate of cancer patients undergoing pericardiocentesis could have several potential explanations. First, pericardiocentesis is often performed in the sicker cancer population. For example, cardiac tamponade is a strong indication for therapeutic pericardiocentesis but is more often present in sicker patients with the higher risk profile.^3^ Similarly, patients undergoing diagnostic pericardiocentesis such as those with undiagnosed pre‐existent cancer or those with ambiguous cancer disease (uncertain primary site) are commonly late presenters with advanced cancer stage with metastasis.^19^ Therefore, it is possible that pericardiocentesis in cancer patients simply indicates sicker patients with a higher risk profile. Second, pericardiocentesis could be associated with serious complications such as arterial and cardiac injury, solid organ injury, hydropneumothorax, arrhythmias, infection and bleeding, even when performed by experts in a controlled environment.^7^ For example, El Haddad et al. reported major procedural complications in five patients and minor procedural complications in 72 patients out of 212 cancer patients undergoing pericardiocentesis.^5^ Although usually reversible and not associated with a fatal outcome, these complications represent a substantial burden to this high‐risk population.^5^ Nevertheless, pericardiocentesis was shown to be a safe procedure in cancer patients in the hospital setting, even in those with thrombocytopenia.^5^, ^8^ It is, therefore, most likely that other cancer‐related and patient‐related factors affect the mortality outcome, and not the procedure itself.

This study distinguished different predictors of increased mortality with pericardiocentesis. Interestingly, there was no association between age and mortality in this setting, highlighting the importance of other patient risk factors such as metastatic status, frailty (weight loss) and haemostatic capacity. Metastatic status is a well‐known unfavourable prognostic factor in cancer patients undergoing pericardiocentesis.^6^, ^8^ Weight loss is an important indicator of more advanced disease, as well as a strong measure of patient frailty. Previous studies have shown that weight loss is associated with a worse prognosis in cancer patients.^20^ The present analysis detected a significant association between weight loss and all‐cause mortality which is consistent with the findings in the overall cancer cohort.^20^ Coagulopathy was also associated with increased mortality in this study, highlighting the importance of secondary haemostasis for the safe performance of invasive procedures such as pericardiocentesis. Previous studies suggested that thrombocytopenia was associated with worse outcomes,^5^ and it was even considered a contraindication for pericardiocentesis,^10^ but other studies have not shown any association with mortality after multivariable adjustment.^8^ Similarly, our study shows thrombocytopenia is not a predictor of increased mortality in cancer patients who underwent pericardiocentesis.

Interestingly, patients undergoing pericardiocentesis without cardiac tamponade had even worse mortality in most cancer types. This could be potentially explained by lower effusion volume and a probably higher proportion of diagnostic indications for pericardiocentesis in this subpopulation. Additionally, due to low effusion volume in patients without cardiac tamponade the risk of cardiac, surrounding vascular and lung injury is high due to technical difficulty leading to higher mortality. This could highlight the importance of proper non‐invasive cancer assessment and utilisation of invasive procedures only in selected cases. However, the design of this study does not allow for such detailed analysis and further studies should re‐assure these speculations.

Clinical implications of the study include the delineation of the most common cancer types undergoing pericardiocentesis and predictors of increased mortality. This study could potentially support usual echocardiographic assessment and cardiology follow‐up in patients with specific cancer types. Bearing in mind the observed increased mortality in the cohort undergoing pericardiocentesis, our data support increased utilisation of preventive measures (ultrasound‐guided puncture, careful preparation and planning, performance by experienced team members and close follow‐up).

There are several limitations of this study. Potential coding issues associated with databases such as the NIS represent an inherent limitation of this study. It was not possible to differentiate if the pericardiocentesis procedure was done for diagnostic or therapeutic purposes, as well as the timing of cancer diagnosis (known cancer vs. newly diagnosed cancer). Furthermore, the transition between ICD‐9 and ICD‐10 systems could have affected the captured estimates. Similarly, an inadequate granularity of the ICD‐9 coding system did not allow for the detection of important subpopulations such as overall patients with pericardial effusion, or those undergoing pericardial window procedure. The observational nature of the study allows for the determination of association, but not a causal relationship. The study results are limited to the in‐hospital period and longer‐term outcomes were not assessed. NIS does not track recurrent procedures and readmissions which could be important for this population. The study was unable to assess direct procedural outcomes such are procedure‐related bleeding or other inadvertent events. The NIS does not contain data on the laboratory and detailed clinical parameters which precludes further analyses. Similarly, it was not possible to include detailed data on cancer treatment or grading some patient factors such as thrombocytopenia and anaemia (mild to severe), as well as renal failure (Stages 1–5). Finally, cancer‐related factors such as cancer activity, cancer staging, cancer duration or performance status measures (e.g., Eastern Cooperative Oncology Group Performance Status) are not available with the NIS.

In conclusion, pericardiocentesis is an infrequent procedure in cancer patients that is most commonly performed in patients with lung and bronchus, haematological, breast, heart and mediastinum, gastroesophageal and female genital cancer. When performed, it is associated with substantially increased all‐cause mortality, irrespectively of the underlying cancer type. Further longitudinal studies are necessary to delineate particular differences amongst cancer types and long‐term outcomes associated with pericardiocentesis.

## AUTHOR CONTRIBUTIONS

**Andrija Matetic:** Conceptualization (equal); formal analysis (lead); methodology (equal); software (lead); visualization (lead); writing – original draft (lead); writing – review and editing (equal). **Bonnie Ky:** Methodology (supporting); supervision (supporting); writing – review and editing (equal). **Eric H. Yang:** Methodology (supporting); supervision (supporting); writing – review and editing (equal). **Phyo K. Myint:** Methodology (supporting); supervision (supporting); writing – review and editing (equal). **Muhammad Rashid:** Methodology (supporting); resources (equal); supervision (supporting); writing – review and editing (equal). **Shelley Zieroth:** Methodology (supporting); supervision (supporting); writing – review and editing (equal). **Timir K. Paul:** Methodology (supporting); supervision (supporting); writing – review and editing (equal). **Ayman Elbadawi:** Methodology (supporting); supervision (supporting); writing – review and editing (equal). **Mamas A. Mamas:** Conceptualization (lead); methodology (lead); resources (lead); supervision (lead); writing – original draft (equal); writing – review and editing (lead).

## FUNDING INFORMATION

None.

## CONFLICT OF INTEREST

The authors declare that there is no conflict of interest.

## Supporting information


Figure S1

Figure S2

Figure S3

Table S1

## Data Availability

The data underlying this article will be shared on reasonable request to the corresponding author.

## Appendix A APPENDICES

STrengthening the Reporting of OBservational studies in Epidemiology (STROBE) statement (attached separately).
