# Supplementary figures and images for: Prevalence, characteristics and mortality of cancer patients undergoing pericardiocentesis in the United States between 2004 and 2017
Source: Cancer Med. 2022 Oct 20;12(5):5471–84. doi: 10.1002/cam4.5373 (PMC10028040; doi:10.1002/cam4.5373)

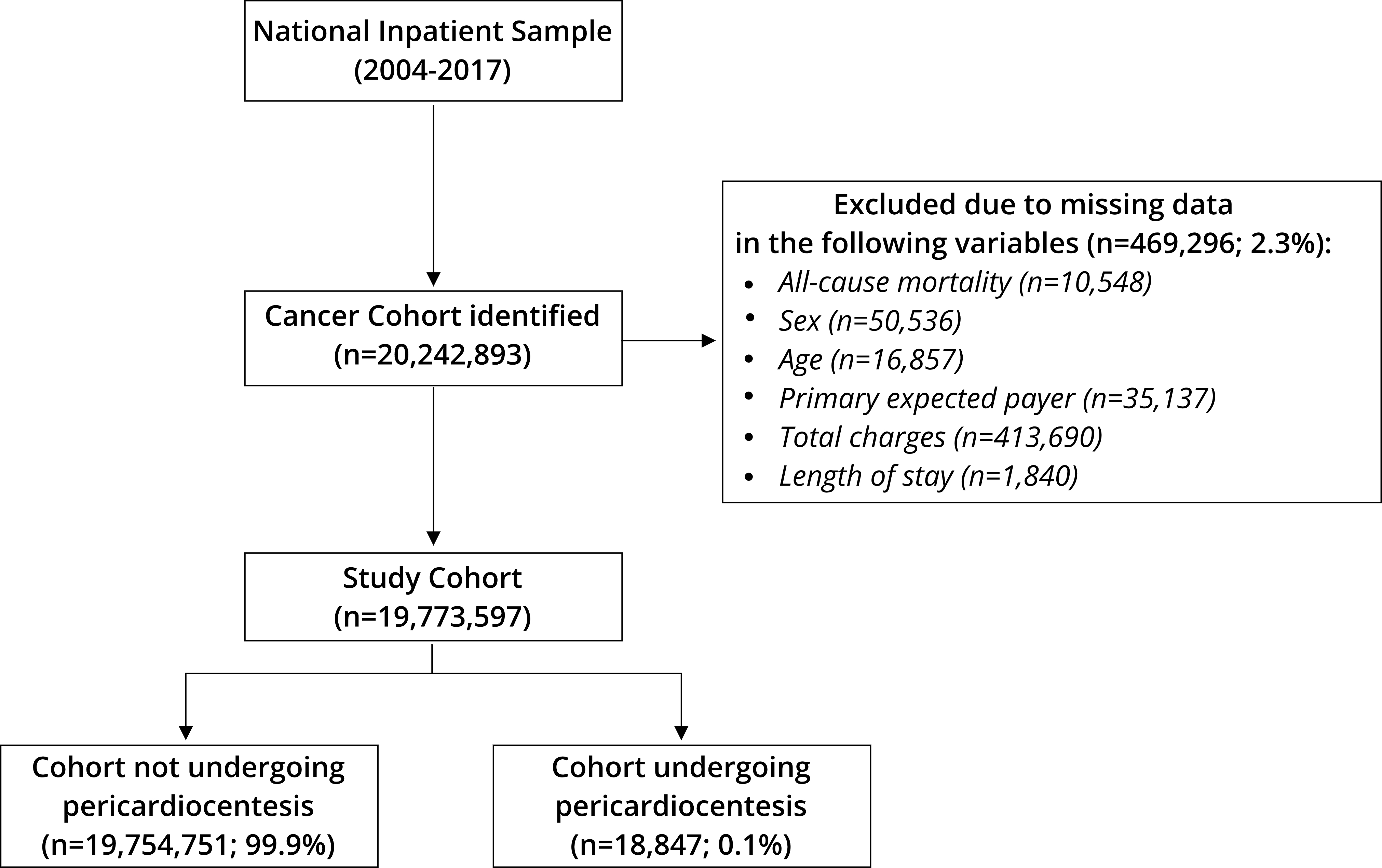

Supplement: Supplementary file 1 — Figure S1 [file CAM4-12-5471-s002.jpg]

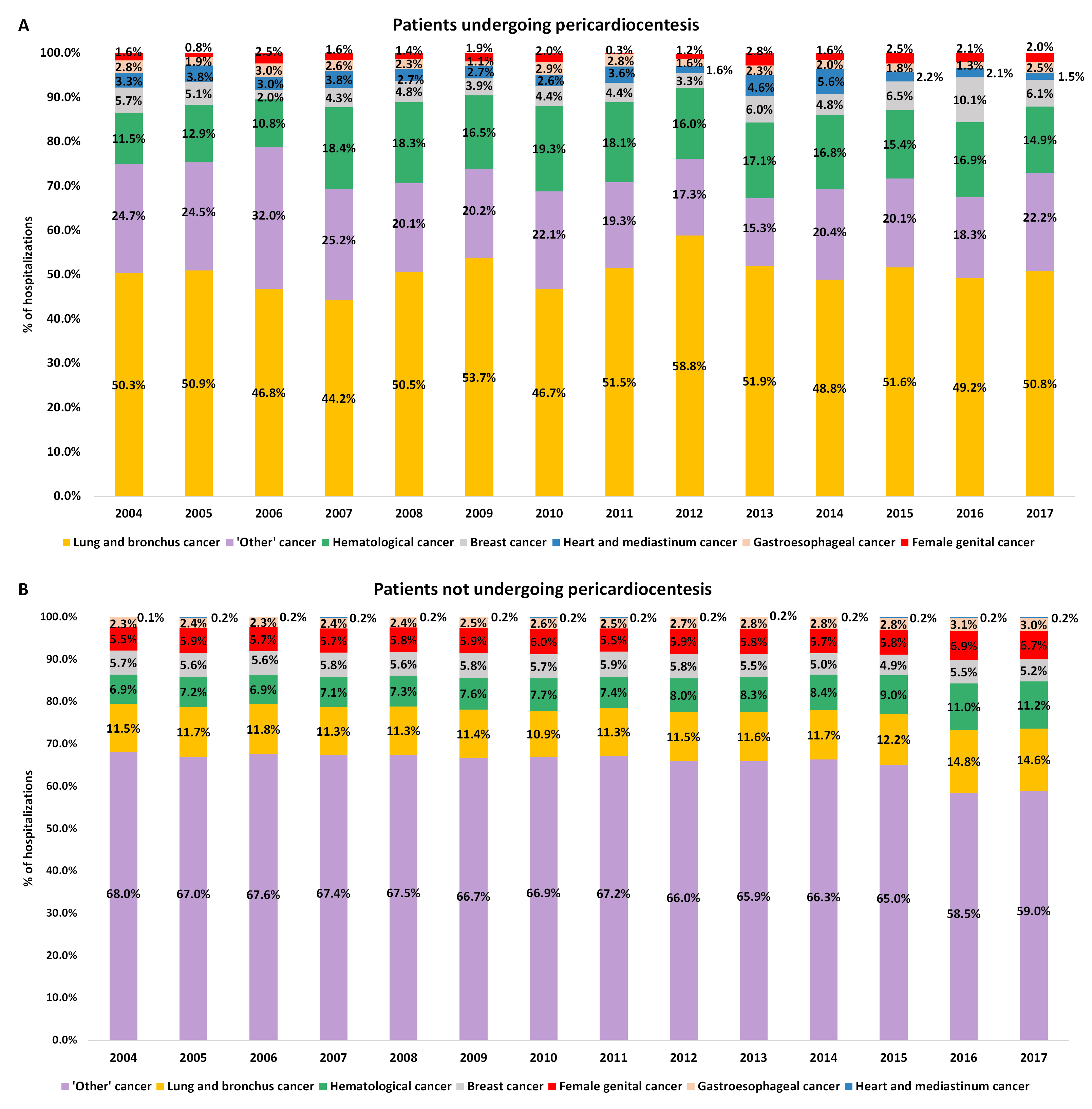

Supplement: Supplementary file 2 — Figure S2 [file CAM4-12-5471-s004.TIF]

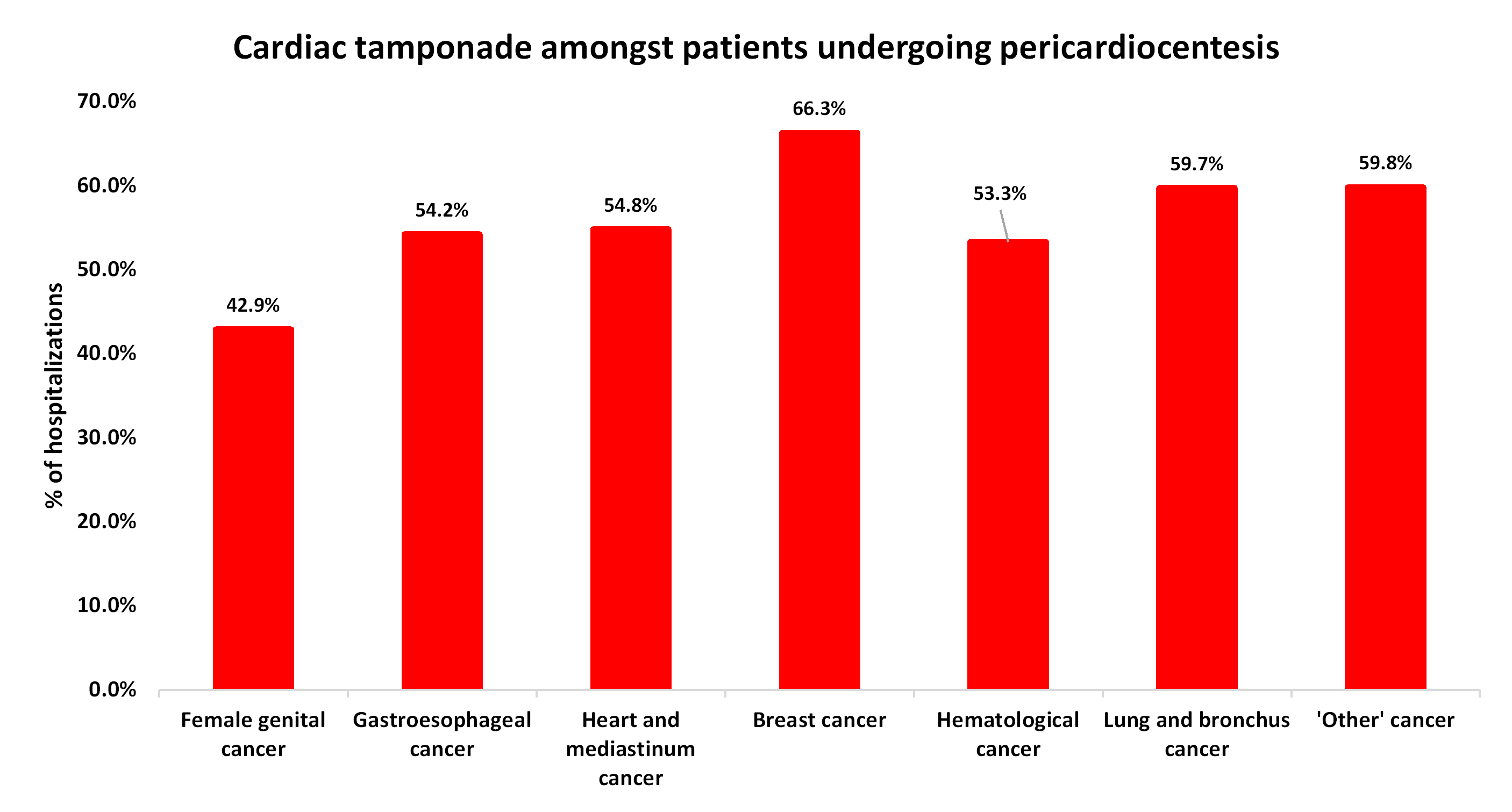

Supplement: Supplementary file 3 — Figure S3 [file CAM4-12-5471-s003.TIF]
