# Supplementary material for: Prevalence, characteristics and mortality of cancer patients undergoing pericardiocentesis in the United States between 2004 and 2017
Source: Cancer Med. 2022 Oct 20;12(5):5471–84. doi: 10.1002/cam4.5373 (PMC10028040; doi:10.1002/cam4.5373)
Supplement: Supplementary file 4 — Table S1 [file CAM4-12-5471-s001.docx]

**Supplementary Tables**

**Supplementary Table 1.** Search codes.

| **Diagnoses** | **ICD-9 Codes (NIS 2004-2015Q3)** | **ICD-10 Codes (NIS Q42015-2017)** |
| --- | --- | --- |
| **Cancer** | 140-209 | C00-C96 |
| **Colorectal cancer** | 153, 154 | C18-C21 |
| **Breast cancer** | 174, 175 | C50 |
| **Lung and bronchus cancer** | 162 | C34 |
| **Hematological cancer** | 200-208 | C81-C96 |
| **Renal and urinary cancer** | 188-189 | C64-C68 |
| **Female genital cancer** | 179-184 | C51-C58 |
| **Male genital cancer** | 185-187 | C60-C63 |
| **Pancreatic cancer** | 157 | C25 |
| **Gastroesophageal cancer** | 150-151 | C15-C16 |
| **Bone cancer** | 170 | C40-C41 |
| **Skin cancer** | 172-173 | C43-C44 |
| **Malignant neoplasm of heart, mediastinum and pleura** | I63-164 | C38; C45.0 |
| **Connective and other soft tissue** | 171 | C49 |
| **Cancer of thyroid and other glands** | 193-194 | C73-C75 |
| **Atrial Fibrillation/flutter** | 427.31 | I48 |
| **Diabetes Mellitus** | 648.0.x, 249.x, 250.x | E08* E09* E10* E11* E13* |
| **Heart failure** | 428.x | I50* Cardiomyopathy: I42* |
| **Thrombocytopenia** | 287.5, 287.49 | D69.4*, D69.5*, D69.6* |
| **Dementia** | 290.x, 294.x | F01*, F02*, F03* |
| **Valvular disease** | 093.2, 394.x-397.1, 397.9, 424.x, 746.3-746.6, V42.2, V43.3 (Elixhauser comorbidity codes) | I34*, I35*, I36*, I37* |
| **Peripheral vascular disorders** | 440.x, 441.x, 442.x, 443.1-443.9, 447.1, 557.1, 557.9, V43.4 (Elixhauser comorbidity codes) | I73* |
| **Arterial hypertension** | 401.1, 401.9, 642.0, 401.0, 402.x-405.x, 642.1,  642.2, 642.7, 642.9 (Elixhauser comorbidity codes) | I10* |
| **Chronic pulmonary disease** | 490x-492.x, 493.x, 494x-505.x, 506.4 (Elixhauser comorbidity codes) | J41*, J42*, J43*, J44*, J45*, J47* |
| **Hypothyroidism** | 243-244.2, 244.8, 244.9 (Elixhauser comorbidity codes) | E03.x |
| **Renal failure** | 403.01, 403.11, 403.91, 404.02, 404.03, 404.12, 404.13, 404.92, 404.93, 585.x, 586.x, V42.0, V45.1, V56.x (Elixhauser comorbidity codes) | N18* |
| **Liver disease** | 070.22, 070.23, 070.32, 070.33, 070.44, 070.54,  456.0, 456.1, 456.20, 571.0, 571.2-571.9, 572.3, 572.8, V42.7 (Elixhauser comorbidity codes) | K70*, K72.1*, K72.9*, K73*, K74*, K75*, K76*, K77* |
| **AIDS** | 042.x-044.x (Elixhauser comorbidity codes) | B20 |
| **Metastatic cancer** | 196.x-199.x (Elixhauser comorbidity codes) | C77*, C78*, C79*, R18.0*, C7B* |
| **Coagulopathy** | 286.x, 287.1, 287.3-287.5 (Elixhauser comorbidity codes) | D65, D66, D67, D68*, D69* |
| **Obesity** | 278.0 (Elixhauser comorbidity codes) | E65, E66.x |
| **Fluid and electrolyte disorders** | 276.x (Elixhauser comorbidity codes) | E22.2, E86.x, E87.x |
| **Anemias** | 280.0, 648.2, 280.1-281.9, 285.2, 285.9 (Elixhauser comorbidity codes) | D62*, D63*, D64* |
| **Alcohol abuse** | 291.0-291.3, 291.5, 291.8, 291.9, 303.x, 305.0 (Elixhauser comorbidity codes) | F10, G62.1, I42.6, K29.2, K70.0, K70.3, K70.9, Z50.2, Z71.4, Z72.1 |
| **Drug abuse** | 292.0, 292.82-292.89, 292.9, 304.x, 305.2-305.9, 648.3 (Elixhauser comorbidity codes) | F11.x-F16.x, F18.x, F19.x, Z71.5. Z72.2 |
| **In-hospital procedures and outcomes** | |  |
| **Procedure-related haemorrhage** | 998.11 | Complicating CA or PCI: I97.410 and I97.610; Complicating CABG: I97.411 and I97.611 |
| **Major bleeding** | 430, 431, 432x, 578x, 786.3, 786.30, 786.39 | I60*, I61*, I62*, R58, K92.0, K92.1, K92.2 |
| **Cardiac tamponade** | 423.3 | I31.4 |
| **Hemopericardium** | 423.0 | I31.2 |
| **Pericardiocentesis** | 37.0 | 0W9D3, 0W9D4 |

**Legend:** CABG – coronary artery bypass grafting; CVA – cerebrovascular accident; IABP – intraaortic balloon pump; IHD – ischemic heart disease; MI – myocardial infarction; NSTEMI – non ST-elevation myocardial infarction; PCI – percutaneous coronary intervention; STEMI – ST-elevation myocardial infarction; TIA – transient ischemic attack.
